# Partial Replacement with Menhaden Oil Improves Peripheral Neuropathy in High-Fat-Fed Low-Dose Streptozotocin Type 2 Diabetic Rat

**DOI:** 10.1155/2012/950517

**Published:** 2012-08-21

**Authors:** Lawrence J. Coppey, Amey Holmes, Eric P. Davidson, Mark A. Yorek

**Affiliations:** ^1^Department of Internal Medicine, University of Iowa, Iowa City, IA 52246, USA; ^2^Iowa City VA Health Care System, U.S. Department of Veterans Affairs, Iowa City, IA 52246, USA

## Abstract

*Aims*. To determine the effect of partial replacement of a high-fat diet with menhaden oil on diabetic neuropathy in an animal model of type 2 diabetes. *Materials and Methods*. High-fat/low-dose streptozotocin diabetic rats were used to examine the influence of replacing 50% of the source of the high-fat diet (lard) with menhaden oil, a natural source of n-3 fatty acids, on diabetic neuropathy. Endpoints included analyses of glucose tolerance, fatty liver disease, serum and liver fatty acid composition, serum lipid and adiponectin levels, motor and sensory nerve conduction velocity, thermal sensitivity and innervation of the hindpaw. *Results*. Diabetic rats were insulin resistant and menhaden oil did not improve whole animal glucose utilization. Menhaden oil did not improve elevated HbA_1_C levels or serum lipid levels but serum levels of adiponectin were significantly increased and hepatic steatosis was significantly improved. Diabetic rats were thermal hypoalgesic, had reduced motor and sensory nerve conduction velocities and intraepidermal nerve fiber profiles were decreased in the hindpaw and these endpoints were significantly improved with menhaden oil. *Conclusions*. We found that enrichment of a high-fat diet with menhaden oil improved a number of endpoints associated with diabetic neuropathy.

## 1. Introduction

It is generally accepted that increased consumption of n-3 fatty acids lowers the risk of cardiovascular disease [1]. The main source of n-3 fatty acids in the Western diet is fish, especially oily fish [1]. Over several decades a large number of studies have found an inverse association between fish consumption and morbidity and mortality from coronary heart disease [1–3]. Blood levels of n-3 fatty acids also appear to correlate inversely with death from cardiovascular causes [1, 4, 5]. Less is known about the effects of n-3 fatty acids on diabetic complications such as neuropathy. One group of investigators has found that fish oil supplementation prevented diabetes-induced nerve conduction velocity and neuroanatomical changes in rats [6]. In contrast, this and another group has found that n-6 fatty acid treatment of diabetic rats was more effective than treatment with n-3 fatty acids on preventing impaired peripheral nerve function in diabetic rats [7–10]. These studies were performed using streptozotocin-induced type 1 diabetic rats. In obese Zucker rats, animal model for metabolic syndrome, a 10% menhaden oil containing diet did not alter obesity or improve insulin resistance [11]. In contrast, in high sucrose-fed rats treatment with menhaden oil prevented but did not reverse insulin resistance [12]. In patients with type-2-diabetes long-term treatments with eicosapentaenoic acid, an n-3 fatty acid had beneficial effects on diabetic neuropathy [13].

 Because of the contrasting results obtained in studies with type 1 diabetic rats and lack of information of the effect of n-3 fatty acid treatment on neuropathy in type 2 diabetic rats we performed studies examining changes in neuropathy as the primary endpoints in high-fat-fed/low-dose streptozotocin-treated rats treated with or without menhaden oil using an intervention protocol. The high-fat-fed/low-dose streptozotocin diabetic rats are an animal model for type 2 diabetes [14, 15]. Rats fed a high-fat diet do not become hyperglycemic presumably due to compensatory hyperinsulinemia [14]. However, treating high-fat-fed rats with a low dose of streptozotocin damages insulin producing *β*-cells so that hyperglycemia develops even though insulin levels are similar or even higher than in chow-fed normoglycemia rats [14]. The diabetes in these rats is analogous to the development of human type 2 diabetes when the decline in hyperinsulinemia is not able to compensate for insulin resistance and hyperglycemia occurs [14]. In our hands this rat models late-stage type 2 diabetes and we were the first to characterize diabetic neuropathy in this model [16].

## 2. Materials and Methods

 Unless stated otherwise all chemicals used in these studies were obtained from Sigma Chemical Co. (St. Louis, MO).

### 2.1. Animals

Male Sprague-Dawley (Harlan Sprague Dawley, Indianapolis, IN) rats 10-11 weeks of age were housed in a certified animal care facility and food (Harlan Teklad, #7001, Madison, WI) and water were provided ad libitum. All institutional (approval ACURF #1202032) and NIH guidelines for use of animals were followed. At 12 weeks of age the rats were separated into three groups. Two of these groups were placed on a high-fat diet (D12451 (45% kcal as fat, 4.7 kcal/g); Research Diets, New Brunswick, NJ). The high-fat diet contained 24 gm% fat, 24 gm% protein, and 41 gm% carbohydrate. The primary source of the increased fat content in the diet was lard. The remaining group was maintained on the control diet (Harlan Teklad, #7001, 3.0 kcal/g, Madison, WI), which contained 4.25 gm% fat. Rats were maintained on the high-fat diet for 8 weeks. Afterwards, these rats were treated with streptozotocin (30 mg/kg in 0.9% NaCl. i.p.). Diabetes was verified 96 h later by evaluating blood glucose levels with the use of glucose-oxidase reagent strips (Aviva Accu-Chek, Roche, Mannheim, Germany). Rats having blood glucose level of 300 mg/dL (11.1 mM) or greater were considered to be diabetic. These rats were maintained on the high-fat diet for an additional 4 weeks. Afterwards, one of the diabetic groups was placed on the high-fat diet with 50% of the fat source replaced with menhaden oil (4.7 kcal/g). The other group of diabetic rats remained on the standard high-fat diet. The rationale for this design was that we wanted to examine the effect of partial substitution of a diet high in saturated fat with a diet enriched with polyunsaturated fat derived from fish oils. Most previous studies have tested the effect of diets enriched with saturated fats versus polyunsaturated fats. It is unlikely that physiologically a patient would completely change diet from containing only saturated fats to a diet containing only polyunsaturated fats. The fatty acid composition of the control diet, standard high-fat diet and the high-fat diet supplemented with menhaden oil are provided in Table 1. These diets were maintained for 16 weeks. Data in Table 2 show the fatty acid composition of the serum of control rats, diabetic rats, and diabetic rats treated with menhaden-oil-supplemented diet. Compared to serum from control rats the stearic acid content is decreased in both diabetic rats and diabetic rats treated with menhaden-oil-supplemented diet. Compared to control and diabetic rats supplementing the diet with menhaden oil causes an increase in eicosapentaenoic acid and docosahexaenoic acid levels and a decrease in arachidonic acid levels.

### 2.2. Glucose Tolerance and Insulin-Stimulated Glucose Uptake by Isolated Soleus Muscle

 Glucose tolerance was determined by injecting rats with a saline solution containing 2 g/kg glucose, i.p., after an overnight fast. Rats were briefly anesthetized with isoflurane and the glucose solution was injected. Immediately prior to the glucose injection and at 15, 30, 45, 60, 120, 180, and 240 min blood samples from the tip of the tail were taken to measure circulating glucose levels using glucose oxidase reagent strips.

### 2.3. Thermal Nociceptive Response

 Thermal nociceptive response in the hindpaw was measured using the Hargreaves method as previously described [17]. Briefly, the rat was placed in the observation chamber on top of the thermal testing apparatus and allowed to acclimate to the warmed glass surface (30°C) and surroundings for a period of 15 min. The mobile heat source was maneuvered so that it was under the heal of the hindpaw and then activated, a process that activates a timer and locally warms the glass surface; when the rat withdrew its paw, the timer and the heat source were turned off and the time was recorded. The timer was defaulted to go off after 25 sec to avoid injury to the rat. Following an initial recording, which was discarded, two measurements were made for each hindpaw, with a rest period of 5 min between each measurement. The mean of the measurements reported in sec were used as the thermal nociceptive response.

### 2.4. Motor and Sensory Nerve Conduction Velocity

 On the day of terminal studies rats were weighed and anesthetized with Nembutal i.p. (50 mg/kg, i.p., Abbott Laboratories, North Chicago, IL). Motor nerve conduction velocity (MNCV) was determined as previously described using a noninvasive procedure in the sciatic-posterior tibial conducting system [18]. The left sciatic nerve was stimulated first at the sciatic notch and then at the Achilles tendon. Stimulation consisted of single 0.2 ms supramaximal (8 V) pulses through a bipolar electrode (Grass S44 Stimulator, Grass Medical Instruments, Quincy, MA). The evoked potentials were recorded from the interosseous muscle with a unipolar platinum electrode and displayed on a digital storage oscilloscope (model 54600A, Hewlett Packard, Rolling Meadows, IL). MNCV was calculated by subtracting the distal from the proximal latency measured in milliseconds from the stimulus artifact of the take-off of the evoked potential and the difference was divided into the distance between the 2 stimulating electrodes measured in millimeters using a vernier caliper; sensory nerve conduction velocity (SNCV) was determined using the digital nerve as described by Obrosova et al. [19]. Briefly, hindlimb SNCV was recorded in the digital nerve to the second toe by stimulating with a square-wave pulse of 0.05 ms duration using the smallest intensity current that resulted in a maximal amplitude response. The sensory nerve action potential was recorded behind the medial malleolus. Sixteen responses were averaged to obtain the position of the negative peak. The maximal SNCV was calculated by measuring the latency to the onset/peak of the initial negative deflection and the distance between stimulating and recording electrodes. The MNCV and SNCV were reported in meters per second.

### 2.5. Intraepidermal Nerve Fiber Density in the Hindpaw

 Immunoreactive intraepidermal nerve fiber profiles, which are primarily sensory nerves, were visualized using confocal microscopy. Samples of skin of the right hindpaw were fixed, dehydrated, and embedded in paraffin. Sections (7 *μ*m) were collected and immunostained with anti-PGP9.5 antibody (rabbit anti human, AbD Serotic, Morpho Sys US Inc., Raleigh, NC) over night followed by treatment with secondary antibody Alexa Fluor 546 goat anti rabbit (Invitrogen, Eugene, OR). Profiles were counted by two individual investigators that were blinded to the sample identity. All immunoreactive profiles within the epidermis were counted and normalized to epidermal length [20, 21].

### 2.6. Biological and Oxidative Stress Markers

Nonfasting blood glucose was determined. Hemoglobin A_1_C levels were determined using a Glyco-tek affinity column kit (Helena Laboratories, Beaumont, TX). Serum samples were collected for determination of free fatty acid, triglyceride, free cholesterol, and adiponectin using commercial kits from Roche Diagnostics, Mannheim, Germany; Sigma Chemical Co., St. Louis, MO; Bio Vision, Mountain View, CA; ALPCO, Salem, NH, respectively. Serum thiobarbituric acid reactive substances levels were determined as a marker of oxidative stress as previously described [22]. Briefly, 200 *μ*L of serum was boiled in 0.75 mL of phosphoric acid (0.19 M), 0.25 mL thiobarbituric acid (0.42 mM), and 0.3 mL water for 60 min. Afterwards, the samples were precipitated with methanol/NaOH and centrifuged for 5 min. The supernatant was measured fluorometrically at excitation wavelength of 532 nm and emission wavelength of 553 nm. Standards were prepared by the acid hydrolysis of 1,1,3,3-tetraethoxypropane. The data was reported as *μ*g/mL serum. Liver and serum samples were collected for analysis of fatty acid composition and triglyceride deposition in liver. For the latter liver samples were embedded in Tissue-Tek O.C.T. compound (Sakura Finetek, Torrance, CA), sectioned (10 *μ*m thickness) and stained with oil red O to determine area of triglyceride deposition in each group by NIH Image. For fatty acid composition determination lipids were extracted from diets, liver, and serum with a 2 : 1 (vol/vol) mixture of chloroform and methanol followed by phase separation with a solution of 154 mM NaCl and 4 mM HCl. Fatty acid composition was measured after the lipid fraction was transesterified in 14% boron trifluoride in methanol and the fatty acid methyl esters extracted into heptane before separation by gas-liquid chromatography [23, 24]. Individual fatty acids peaks as % of total fatty acids present were identified by comparison to known fatty acid standards.

### 2.7. Data Analysis

 Results are presented as mean ± S.E.M. Comparisons between the treatment group and control and nontreated diabetic rats were conducted using one-way ANOVA and Bonferroni *after* test comparison (Prism software; GraphPad, San Diego, CA). A *P* value of less than 0.05 was considered significant.

## 3. Results

### 3.1. Effect of Treatment of High-Fat/Streptozotocin Diabetic Rats with a Menhaden-Oil-Supplemented Diet on Weight and Blood Glucose

Data in Table 3 demonstrate that nontreated and treated diabetic rats gained weight similar to control rats. In diabetic rats treated with menhaden oil there was a trend toward these rats weighing less than control or nontreated diabetic rats but this was not significantly different. Mass of the epididymal fat pad was significantly greater in diabetic rats compared to control rats and this was corrected with menhaden oil treatment (Table 3). All diabetic rats were hyperglycemic at the end of the study period as indicated by significantly elevated nonfasting blood glucose and hemoglobin A_1_C levels (Table 3). Treatment of diabetic rats with menhaden oil did not significantly change blood glucose levels compared to untreated diabetic rats.

### 3.2. Effect of Treatment of High-Fat/Streptozotocin Diabetic Rats with a Menhaden-Oil-Supplemented Diet on Serum Lipid and Thiobarbituric Acid Reactive Substances Levels

Data in Table 4 demonstrate that serum thiobarbituric acid reactive substances, a marker for oxidative stress, were significantly increased in diabetic rats. Treating diabetic rats with a menhaden oil enriched high-fat diet did not improve oxidative stress as determined with this marker. Diabetes caused a significant increase in serum triglycerides, free fatty acids, and cholesterol levels (Table 4). Serum hyperlipidemia was not improved by treating diabetic rats with a menhaden-oil-supplemented diet. In contrast, treating diabetic rats with menhaden-oil-supplemented diet increased serum adiponectin levels compared to control and diabetic rats.

### 3.3. Effect of Treatment of High-Fat/Streptozotocin Diabetic Rats with a Menhaden-Oil-Supplemented Diet on Liver Fatty Acid Composition and Fatty Liver Disease

**Figure 1:**
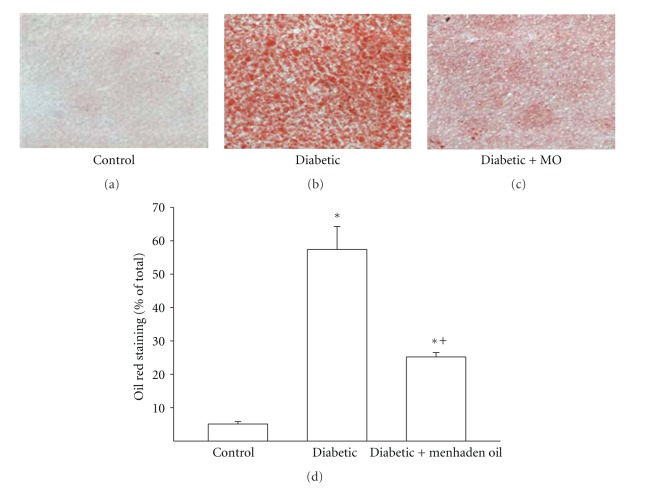
Effect of treatment of high-fat/streptozotocin diabetic rats with menhaden-oil- (MO-) supplemented diet on fatty liver disease. Fat accumulation by the liver was determined using oil red staining. Representative images are provided and data are presented as the mean ± S.E.M in % of total area. The number of rats in each group was the same as shown in Table 2. *P < 0.05 compared to control rats; ^+^ *P* < 0.05 compared to diabetic rats.

Data in Table 5 demonstrate that palmitic and stearic acid are significantly increased and oleic acid significantly decreased in liver from diabetic rats compared to control rats. Feeding diabetic rats a high-fat diet enriched with menhaden oil caused a significant increase in eicosapentaenoic and docosahexaenoic acid and a significant decrease in oleic and arachidonic acid in the liver compared to control rats. Compared to nontreated diabetic rats menhaden oil treatment of diabetic rats caused a significant increase in liver eicosapentaenoic and docosahexaenoic acid and a significant decrease in palmitic and stearic acid. Data in Figure 1 demonstrate that fatty acid accumulation was significantly increased in diabetic rats and was significantly improved when diabetic rats were treated with a menhaden-oil-enriched high-fat diet. However, fatty acid accumulation in liver from menhaden-oil-treated diabetic rats remained significantly elevated compared to control rats.

### 3.4. Effect of Treatment of High-Fat/Streptozotocin Diabetic Rats with a Menhaden-Oil-Supplemented Diet on Glucose Tolerance

**Figure 2:**
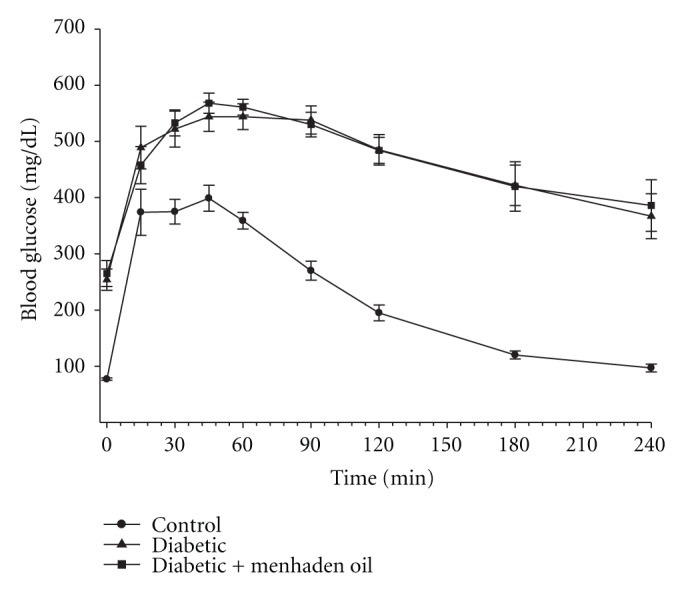
Effect of treatment of high-fat/streptozotocin diabetic rats with menhaden-oil- (MO-) supplemented diet on glucose tolerance. Glucose tolerance was determined as described in Section 2. Data are presented as the mean ± S.E.M. in mg/dL. The number of rats in each group was the same as shown in Table 2.

Glucose utilization was significantly impaired in diabetic rats and this was not improved by treating diabetic rats with a high-fat diet enriched with menhaden oil (Figure 2). 

### 3.5. Effect of Treatment of High-Fat/Streptozotocin Diabetic Rats with a Menhaden-Oil-Supplemented Diet on Nerve Conduction Velocity, Thermal Nociception, and Intraepidermal Nerve Fiber Density

**Figure 3:**
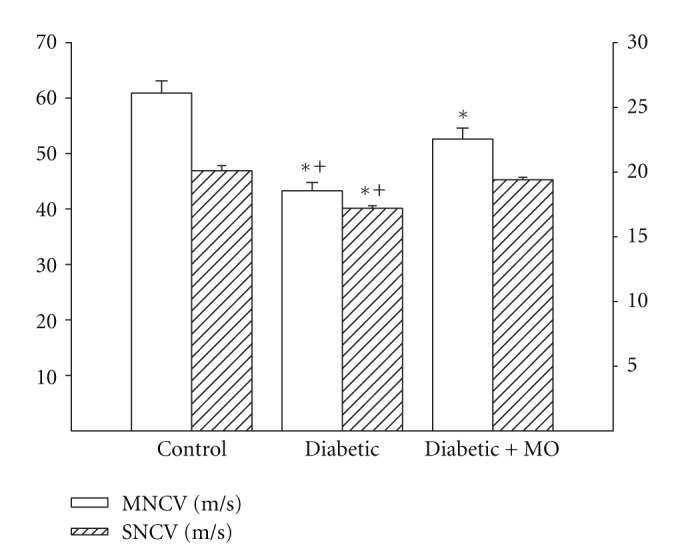
Effect of treatment of high-fat/streptozotocin diabetic rats with menhaden-oil- (MO-) supplemented diet on motor and sensory nerve conduction velocity. Motor and sensory nerve conduction velocity was examined as described in Section 2. Data are presented as the mean ± S.E.M. in m/sec. The number of rats in each group was the same as shown in Table 2. *P < 0.05 compared to control rats; ^+^ *P* < 0.05 compared to diabetic rats.

**Figure 4:**
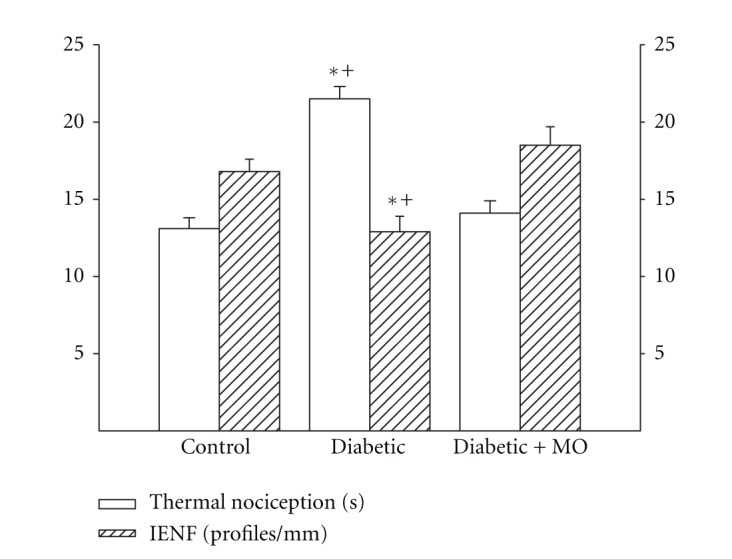
Effect of treatment of high-fat/streptozotocin diabetic rats with menhaden-oil- (MO-) supplemented diet on thermal nociception and intraepidermal nerve fiber density. Thermal nociception and intraepidermal nerve fiber density was examined as described in Section 2. Data are presented as the mean ± S.E.M. for thermal nociception in sec and intraepidermal nerve fiber profiles per mm. The number of rats in each group was the same as shown in Table 2. *P < 0.05 compared to control rats; ^+^ *P* < 0.05 compared to diabetic rats.

Motor and sensory nerve conduction velocity was significantly decreased indiabetic rats compared to control rats (Figure 3). Treating diabetic rats with a high-fat diet enriched with menhaden oil significantly improved motor and sensory nerve conduction velocity compared to diabetic rats although motor nerve conduction in diabetic rats treated with menhaden oil remained significantly decreased compared to control rats (Figure 3). Data in Figure 4 demonstrate that diabetic rats are hypoalgesic to thermal stimuli compared to control rats and this was significantly improved when diabetic rats were treated with a high-fat diet enriched with menhaden oil. Intraepidermal nerve fiber profiles in the hindpaw of diabetic rats were significantly decreased compared to control rats and this was prevented by treating diabetic rats with a high-fat diet enriched with menhaden oil (Figure 4).

## 4. Discussion

 The goal of these studies was to determine whether partial replacement of saturated fats derived from lard in the high-fat diet of high-fat/low-dose streptozotocin diabetic rats with menhaden oil, a natural source of n-3 fatty acids, improves diabetic peripheral neuropathy. Some of the unique features of this study were the design and that high-fat/low-dose streptozotocin diabetic rats, a model for type 2 diabetes, were used. This was an intervention study and to enrich the diet with n-3 fatty acids, menhaden oil was substituted for lard in the high-fat diet. However, only 50% of the calories derived from lard were replaced with menhaden oil. Diabetic rats remained on the lard-derived high-fat diet for 4 weeks after inducement of diabetes and afterwards the treatment group was placed on the menhaden-oil-enriched high-fat diet. Most previous studies of fatty acid enrichment used diets containing a higher percentage of the targeted treatment and were designed as prevention studies. 

 The major findings from this study was that partial replacement of saturated fats derived from lard with n-3-fatty-acid-enriched menhaden oil in the high-fat diet of high-fat-fed/low-dose streptozotocin diabetic rats significantly improved fatty liver disease and several endpoints for peripheral neuropathy. In contrast, impaired glucose utilization was not improved with menhaden oil. It has been reported that eicosapentaenoic acid prevents and reverses insulin resistance in high-fat-fed mice via modulation of adipose tissue inflammation [25]. In ob/ob mice dietary intake of n-3 fatty acids had insulin sensitizing actions in adipose tissue and liver and improved insulin resistance [26]. In contrast, improved n-3 fatty acid status did not improve insulin resistance in fa/fa Zucker rat model [11]. We attribute the lack of effect of menhaden oil treatment on glucose utilization by high-fat-fed/low-dose streptozotocin diabetic rats to the inability of this rat model to produce a sufficient insulin response when challenged with a glucose load. In our hands the high-fat-fed/low-dose streptozotocin diabetic rat best models late-stage type 2 diabetes [16, 27]. 

 One mechanism that could explain some of the beneficial effects of enriching the diet with menhaden oil is reduction in inflammatory stress. In the serum the ratio of arachidonic acid to eicosapentaenoic acid and docosahexaenoic acid was 5 : 7 : 0.6 for control rats, diabetic rats, and diabetic rats treated with menhaden oil supplemented diet. In the liver the results were similar 5 : 4 : 0.6. An increased n-6/n-3 long-chain polyunsaturated fatty acid ratio favors a pro-inflammatory state [28]. This suggests that the potential for inflammatory mediators being produced are significantly reduced in high-fat-fed/low-dose streptozotocin diabetic rats fed the menhaden-oil-supplemented diet. n-3 Fatty acid enrichment is well known to have anti-inflammatory effects and adiponectin is an anti-inflammatory adipokine [29–33]. In these studies replacing 50% of the high-fat diet with menhaden oil caused a significant increase in serum adiponectin levels compared to control and diabetic rats. n-3 Fatty acids have been demonstrated to reverse nonalcoholic steatohepatitis in rats treated with a methionine-/choline-deficient diet by reducing the inflammatory response through lowering the n-6/n-3 fatty acid ratio [34, 35]. Nonalcoholic fatty liver disease is now recognized as the hepatic component of the metabolic syndrome [36]. It has been estimated that nonalcoholic fatty liver disease exists in up to 70% of people with type 2 diabetes [36]. Nonalcoholic fatty liver disease is strongly associated with insulin resistance and often contributes to poor glycemic control [36]. Thus, successful treatment for nonalcoholic fatty liver disease may reduce the risk and improve glycemic control in type 2 diabetes and n-3 fatty acids may be a promising treatment for nonalcoholic fatty liver disease [37]. 

 Metabolites of eicosapentaenoic acid and docosahexaenoic acid referred to as resolvins (resolution-phase interaction products) provide antioxidant, anti-inflammatory, and neuroprotection [38, 39]. Resolvins are oxygenated metabolites of eicosapentaenoic acid (E series resolvins) and docosahexaenoic acid (D series resolvins). In nonvascular tissue 15-lipoxygenase is responsible for the generation of resolvins, and eicosapentaenoic acid and docosahexaenoic acid are good substrates for 15-lipoxygenase. Resolvin formation can be increased by consuming increased amounts of eicosapentaenoic acid or docosahexaenoic acid [38, 39]. Cortina et al. [40] has demonstrated that regeneration of corneal nerves damaged by refractive surgery can be increased with treatment using docosahexaenoic acid. It has also been previously reported that fish oil supplementation prevents nerve conduction velocity and neuroanatomical changes in type 1 diabetic rats [6]. In the current study we found that partial replacement with menhaden oil of saturated fat in the high-fat diet of a rat model of type 2 diabetes improves endpoints associated with diabetic neuropathy. It is possible that enriching the diet of diabetic rats with menhaden oil contributed to an increase in resolvin production and neural protection/regeneration. Future studies will determine the effect menhaden oil supplementation has on reducing oxidative and inflammatory stress and/or promoting the formation of neuroprotective compounds such as resolvin.

 In summary, we have demonstrated that partial replacement of saturated fat with menhaden oil, a natural source of n-3 fatty acids, in the high-fat diet of high-fat-fed/low-dose streptozotocin diabetic rats, a model for type 2 diabetes, improves motor and sensory nerve impairments associated with diabetes. Improvement in diabetic neuropathy endpoints occurred without improvement in insulin resistance. This suggests that dietary enrichment with n-3 fatty acids may be beneficial treatment for diabetic neuropathy.

## Figures and Tables

**Table 1:** Fatty acid % composition of diets measured by gas chromatography.

Diet	16 : 0	18 : 0	18 : 1	18 : 2	20 : 5	22 : 6
Control (3)	24 ± 3	8 ± 1	29 ± 3	30 ± 4	ND	ND
Standard high fat (3)	20 ± 2	10 ± 2	35 ± 4	29 ± 4	ND	ND
50% menhaden oil (3)	18 ± 3	7 ± 1	21 ± 2	15 ± 2	10 ± 2	7 ± 1

Data are presented as the mean ± S.E.M. ND: not detected. Parentheses indicate the number of experimental determinations.

**Table 2:** Effect of partial menhaden oil substitution on fatty acid % composition of serum measured by gas chromatography.

Diet	16 : 0	18 : 0	18 : 1	18 : 2	20 : 4	20 : 5	22 : 6
Control (10)	18.1 ± 1.4	13.1 ± 0.8	9.9 ± 0.9	16.5 ± 0.9	19.8 ± 1.4	0.5 ± 0.3	3.4 ± 0.6
Diabetic (10)	17.6 ± 0.6	19.8 ± 1.0^a^	9.5 ± 0.5	19.4 ± 0.6	20.8 ± 1.0	ND	2.9 ± 0.2
Diabetic + menhaden oil (10)	20.4 ± 0.9	17.1 ± 0.5^a^	8.1 ± 0.5	18.6 ± 0.9	10.0 ± 0.4^a,b^	5.4 ± 0.5^a,b^	10.0 ± 0.8^a,b^

Fatty acid unsaturation index: control 1.57 ± 0.05; diabetic 1.56 ± 0.03; diabetic + menhaden oil 1.78 ± 0.04^a,b^. Data are presented as the mean ± S.E.M. ND: not detected. ^a^ *P* < 0.05 compared to control; ^b^ *P* < 0.05 compared to diabetic. Parentheses indicate the number of experimental determinations.

**Table 3:** Effect of partial menhaden oil substitution in the diet of high-fat/streptozotocin diabetic rats on change in body weight, blood glucose, hemoglobin A_1_C, and epididymal fat pad.

Determination	Control (10)	Diabetic (10)	Diabetic + menhaden oil (10)
Start weight (g)	316 ± 2	306 ± 2	309 ± 2
End weight (g)	505 ± 9	503 ± 16	461 ± 27
Blood glucose (mg/dL)	158 ± 6	351 ± 39^a^	400 ± 37^a^
Hb A_1_C (%)	8.9 ± 0.3	16.6 ± 0.9^a^	14.0 ± 1.0^a^
Epididymal fat pad (g)	6.6 ± 0.4	9.2 ± 0.8^a^	6.6 ± 0.8

Data are presented as the mean ± S.E.M. ^a^ *P* < 0.05 compared to control. Parentheses indicate the number of experimental animals.

**Table 4:** Effect of partial menhaden oil substitution in the diet of high-fat/streptozotocin diabetic rats on serum thio barbituric acid reactive substances, triglycerides, free fatty acids, cholesterol, and adiponectin.

Determination	Control (10)	Diabetic (10)	Diabetic + menhaden oil (10)
Thiobarbituric acid reactive substances (*μ*g/mL)	0.57 ± 0.04	0.87 ± 0.04^a^	0.92 ± 0.11^a^
Triglycerides (mg/dL)	22 ± 6	53 ± 8^a^	114 ± 20^a^
Free fatty acids (mmol/L)	0.07 ± 0.02	0.30 ± 0.05^a^	0.39 ± 0.06^a^
Cholesterol (mg/mL)	0.5 ± 0.2	1.1 ± 0.3^a^	1.9 ± 0.6^a^
Adiponectin (*μ*g/mL)	6.7 ± 0.3	7.1 ± 0.3	9.1 ± 0.5^a,b^

Data are presented as the mean ± S.E.M. ^a^ *P* < 0.05 compared to control; ^b^ *P* < 0.05 compared to diabetic. Parentheses indicate the number of experimental animals.

**Table 5:** Effect of Partial Menhaden Oil Substitution on Fatty Acid % Composition of Liver Measured by Gas Chromatography.

Condition	16 : 0	18 : 0	18 : 1	18 : 2	20 : 4	20 : 5	22 : 6
Control (10)	21.2 ± 0.2	17.3 ± 0.3	9.8 ± 0.3	13.8 ± 0.4	24.8 ± 0.3	0.2 ± 0.1	5.2 ± 0.3
Diabetic (10)	17.0 ± 0.3^a^	23.0 ± 0.3^a^	7.3 ± 0.1^a^	14.6 ± 0.4	25.8 ± 0.5	0.1 ± 0.1	6.5 ± 0.4
Diabetic + Menhaden oil (10)	20.5 ± 0.3^b^	19.4 ± 0.3^b^	6.1 ± 0.1^a^	13.1 ± 0.4	12.5 ± 0.5^a,b^	4.5 ± 0.2^a,b^	17.4 ± 0.7^a,b^

Fatty acid unsaturation index: Control 1.78 ± 0.05; Diabetic 1.86 ± 0.03; Diabetic + Menhaden oil 2.16 ± 0.06^a,b^. Data are presented as the mean ± S.E.M. ^a^ *P* < 0.05 compared to control; ^b^ *P* < 0.05 compared to diabetic. Parentheses indicate the number of experimental animals.
